# Polyethylene Glycol as Shape and Size Controller for the Hydrothermal Synthesis of SrTiO_3_ Cubes and Polyhedra

**DOI:** 10.3390/nano10091892

**Published:** 2020-09-21

**Authors:** Francesco Pellegrino, Fabrizio Sordello, Lorenzo Mino, Marco Prozzi, Ulrich Mansfeld, Vasile-Dan Hodoroaba, Claudio Minero

**Affiliations:** 1 Dipartimento di Chimica and NIS Inter-Department Centre, University of Torino, Via P. Giuria 7, 10125 Torino, Italy; fabrizio.sordello@unito.it (F.S.); lorenzo.mino@unito.it (L.M.); marco.prozzi@unito.it (M.P.); claudio.minero@unito.it (C.M.); 2 JointLAB UniTo-ITT Automotive, Via Quarello 15/A, 10135 Torino, Italy; 3 Federal Institute for Materials Research and Testing (BAM), 12205 Berlin, Germany; Ulrich.Mansfeld@uni-bayreuth.de

**Keywords:** polyethylene glycol, strontium titanate, controlled morphology, photoelectrochemistry

## Abstract

Understanding the correlation between the morphological and functional properties of particulate materials is crucial across all fields of physical and natural sciences. This manuscript reports on the investigation of the effect of polyethylene glycol (PEG) employed as a capping agent in the synthesis of SrTiO_3_ crystals. The crucial influence of PEG on both the shape and size of the strontium titanate particles is revealed, highlighting the effect on the photocurrents measured under UV–Vis irradiation.

## 1. Introduction

Strontium titanate (SrTiO_3_) is a functional material that presents many different and specific physical properties, such as high conductivity (both ionic and electronic), thermoelectricity, strain-induced ferroelectricity, dielectric tunability, etc. [1,2]. Moreover, its applicability in different forms (single crystal, ceramics, thin film, powder) makes it attractive in several application fields. In material science, the control of shape and size has become increasingly important, and several works have been devoted to the study of the morphology effect on a specific functional property [3,4,5,6,7]. SrTiO_3_ has been widely investigated for its use as a photocatalyst for water photo-splitting and for the abatement of organic and inorganic pollutants under irradiation in so-called advanced oxidation processes (AOPs) [8,9]. It is often associated with frequently-studied titanium dioxide (TiO_2_) (usually employed as a starting material for SrTiO_3_ synthesis). However, unlike TiO_2_, it is able to split water without an external electric bias [1,10,11] due to the more suitable band structure of the titanate. SrTiO_3_ is also often doped or coupled with other metals or metal oxides in order to improve its efficiency for H_2_ and O_2_ production under both UV and visible light [12,13,14].

SrTiO_3_ presents a cubic perovskite structure between 110 and 2353 K. At room temperature, the equilibrium shape is a cube in which Sr^2+^ ions are located at the corners of the unit cell, whereas the smaller Ti^4+^ ion is at the center and is 6-fold coordinated to oxide ions located in the centers of the facets [1]. Due to this simple crystal structure, SrTiO_3_ has been studied as a model perovskite.

The synthesis of strontium titanate can be carried out following several methods [15,16,17,18,19]. Among these, hydrothermal synthesis allows the preparation of exceptionally pure crystals with high specific surface area, as well as control of morphology through the variation of synthesis parameters like precursors, temperature, time, pH, shape controllers, etc. [16,20,21,22]. The synthesis of shape and size-controlled crystals, in particular at nanometer and sub-micrometer level, is crucial to understanding the dependence of the functional properties on the morphological characteristics of the particulate material at nanoscale [6,23]. To realize morphological control, capping agents are usually employed, i.e., molecules are added in the synthesis process, and they can be absorbed selectively on specific facets of the crystal, decreasing their surface energy and promoting their growth. Often, the reaction solvent itself acts as a capping agent [19,24,25]. In the hydrothermal synthesis of SrTiO_3_, several shape controllers can be used to finely control the morphology of the particles, including oleic acid, polyvinylpyrrolidone (PVP), glycols, polyols, and alcohols with different pK_a_ [1,19,26]. In this work, different concentrations of polyethylene glycol (PEG) were exploited to control the morphology of SrTiO_3_ particles under hydrothermal conditions, allowing the synthesis of sub-micrometer cuboids and polyhedra with different sizes and shapes. Finally, the implications of the changing morphology on the photoelectrochemical properties of the materials were evaluated by photocurrent experiments.

## 2. Materials and Methods

### 2.1. Reagents

Strontium chloride (hexahydrate, 99+%) and lithium hydroxide (monohydrate, 98%) were purchased from Alfa Aesar (Haverhill, MA, USA). Polyethylene glycol 600 (PEG) and titanium (IV) chloride (99.9%) were obtained from Sigma-Aldrich (St. Louis, MO, USA).

### 2.2. SrTiO_3_ Materials Synthesis

**Figure 1:**
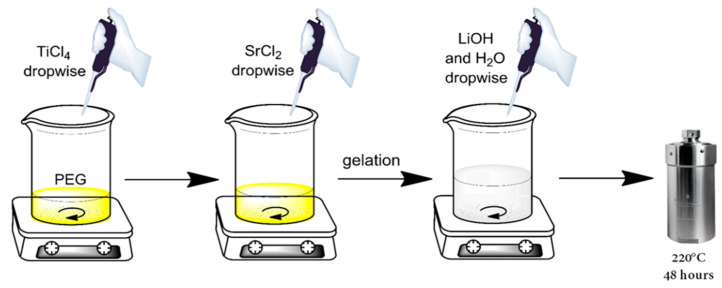
Synthesis scheme of the strontium titanate (SrTiO_3_) particles.

The synthesis of the SrTiO_3_ materials was carried out taking inspiration from the work of Dong and coauthors [26]. In a typical synthesis, the desired amount of PEG was directly weighted in a Teflon vessel at room temperature. The Ti and Sr precursors, TiCl_4_ and SrCl_2_, respectively, were sequentially added dropwise under vigorous stirring. The addition of the SrCl_2_ aqueous solution induced the formation of a white gel (due to the TiCl_4_ hydrolysis) that made agitation difficult. However, the subsequent additions of the base (3 M LiOH) and water allowed for easier stirring. The suspension was stirred for at least 1 h at room temperature for homogenizing, whereupon N_2_ was sparged for 5 min. The final concentration of PEG for each synthesis was 0, 130, 245 and 430 mM, and the initial pH was always above 11. The sealed Teflon vessel was then positioned in a stainless steel autoclave and treated at 220 °C for 48 h. Figure 1 shows the complete procedure for the synthesis of the SrTiO_3_ particles. After the synthesis time, the autoclaves were allowed to cool down to room temperature, and the sub-microparticles (SMPs) were collected and then centrifuged and washed several times against acetone to remove the residual organics. Finally, SMPs were washed twice with Milli-Q water and freeze-dried to obtain the powders. The materials were labeled, indicating the amount, in weight, of the added PEG during the synthesis: SrTIT-0 g, SrTIT-5 g, SrTIT-10 g and SrTIT-20 g.

### 2.3. Materials Characterization

X-ray diffraction (XRD) patterns of the powders were recorded with a Malvern Panalytical X’Pert Pro (Malvern, UK) equipped with an X’Celerator detector powder diffractometer using Cu K radiation generated at 45 kV and 40 mA. The 2 range was from 25° to 100°, with a step size of 0.01° and a counting time of 0.6 s.

Diffuse reflectance (DR) UV–Vis–NIR spectra were acquired using a Varian Cary 5000 (Agilent, Santa Clara, CA, USA) spectrophotometer equipped with an integrating sphere with an inner coating of Spectralon^®^, which was also used as a reference.

The dynamic light scattering (DLS) measurements were carried out using a CILAS Nano DS (Orleans, France) instrument with a scattering angle of 90°. An acidified (HClO_4_) stable suspension (10 mg L^−1^) was analyzed for each sample after sonication for 60 min in an ultrasonic bath in a closed vial to avoid evaporation (suggested 95 W, 37 kHz) and contamination. The reported particle dimensions were obtained through the fit of the (decay time) distribution function to the integral equation, relating the field correlation function and the said distribution function by a constrained regularization method (CONTIN algorithm) developed by Provencher [27]. The intensity distribution function was then obtained.

High-resolution electron micrographs were taken with a Zeiss Supra 40 (Carl Zeiss, Oberkochen, Germany) SEM equipped with an in-lens secondary electron (SE) detector. SEM images were acquired at accelerating voltages of 10 and 20 kV using the in-lens detector. The microscope can be used in the transmission mode, STEM-in-TEM, by preparing the particles on a conventional carbon TEM copper grid. By using a dedicated sample holder, the transmitted electrons are multiplied and collected by the conventional Everhart-Thornley SE detector [28]. Further, energy dispersive X-ray spectroscopy (EDS) with a highly sensitive UltraDry Silicon Drift Detector (SDD) (Thermo Fisher Scientific, Waltham, MA, USA) of a nominal 100 mm^2^ crystal area can be applied in the STEM-in-SEM mode. Hence, spatial resolutions for EDS down to ≈10 nm can be attained [29]. Due to the complex geometry of the sub-µm particles (non-planar surface), an elemental quantification with EDS coupled with the transmission mode is not applicable by the conventional bulk analysis approaches. In this work, TEM images of the materials were obtained using a JEOL 3010 UHR microscope operated at 300 kV. For both types of SEM and TEM measurements, the samples were prepared as dry powders dispersed on a carbon substrate (SEM) and lacey carbon TEM copper grids.

The photoelectrochemical characterization was performed using a standard setup, composed of an Autolab PGSTAT12 computer-controlled potentiostat and a 150 W LOT-Oriel Xe arc lamp as radiation source. The incident irradiance on the sample was 360 W m^−2^ in the 250–400 nm spectral range. The electrochemical cell was a conventional three-electrode cell with a 1 mm thick fused silica window. The counter and reference electrodes were a glassy carbon and an Ag/AgCl/KCl (3 M) electrode, respectively. The working electrode was prepared by dropping a concentrated suspension of the SrTiO_3_ materials (>10 g L^−1^) onto an indium tin oxide (ITO) substrate. The suspension was left to dry in environmental conditions. The amount of deposited SrTiO_3_ was determined by gravimetry. The produced film presented an illuminated area of ~4 cm^2^. Before the measurements, the films were irradiated for at least 2 h in solution (purged with pure O_2_) in order to remove any possible presence of organic residuals that could affect the results. Whereupon, the solution (0.1 M NaClO_4_ and 1 mM HClO_4_, pH 3) was purged with N_2_ for 45 min before each measurement to eliminate the residual O_2_ present in the solution. Photocurrent measurements were then carried out under an N_2_ atmosphere, and an electrical potential was imposed following a progression of 0.5 V from 1 to 2.5 V.

## 3. Results and Discussion

### 3.1. X-ray Diffraction

**Figure 2:**
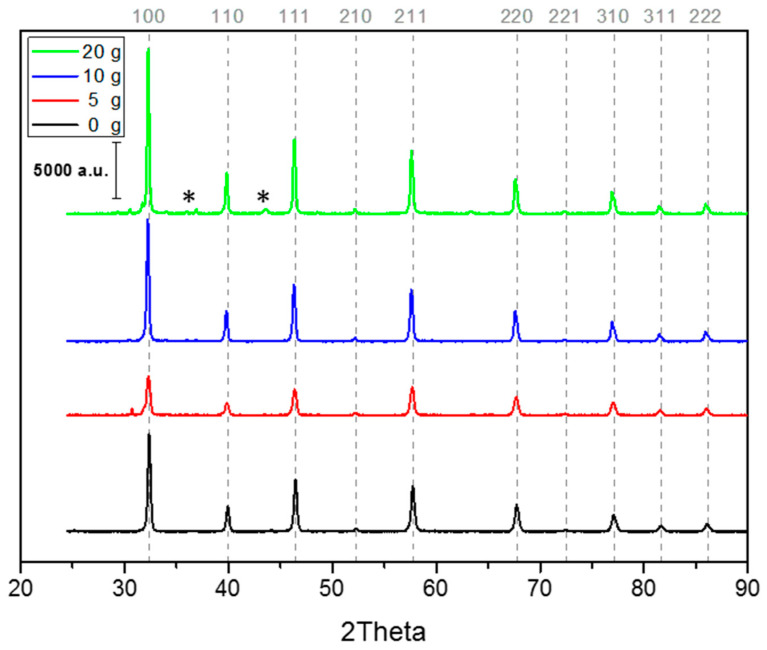
XRD patterns of the four materials synthesized. * Lithiated phase.

XRD data proves that SrTiO_3_ is the main crystalline phase present (Figure 2). Traces of lithiated phase are present in the material SrTIT-20 g; nevertheless, the highest peak of the lithiated phase (2θ = 43°) is 45 times less intense than the 2θ = 33° peak of SrTiO_3_. Therefore, the presence of these traces can be neglected from a morphological point of view, although they may not be overlooked for catalytic applications. Considering the powders obtained with PEG, we observed an increase in the XRD reflex intensities with increasing PEG concentration. The sample SrTIT-5 g has the least intense peaks, even less intense than the control sample SrTIT-0 g, which in turn has less intense peaks than SrTIT-10 g and SrTIT-20 g (Figure 2). Therefore, PEG concentrations of ≤131 mM seem to be deleterious for the crystallization of the materials, and, conversely, PEG concentrations of ≥245 mM improve the crystallinity of the SrTiO_3_ materials. A reference pattern of SrTiO_3_ (JCPDS pattern: 01-089-4934) is reported in Figure S1.

### 3.2. Shape and Size Characterization by Electron Microscopy and Dynamic Light Scattering

**Figure 3:**
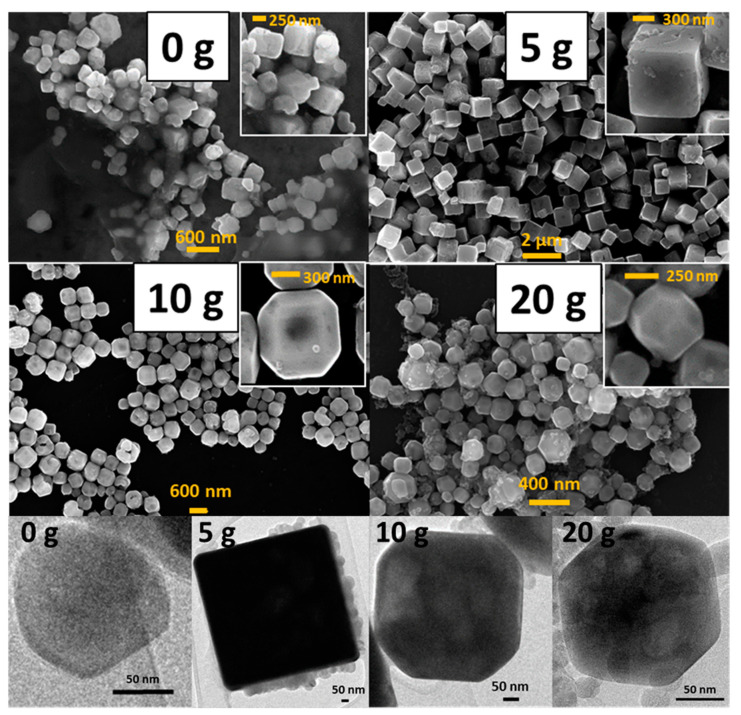
SEM (above) and TEM (below) micrographs of the four synthesized SrTiO_3_ materials.

The synthesized materials were characterized by electron microscopy in order to study the effect of the PEG on their morphology. Figure 3 highlights the strong effect of PEG as the capping agent. Starting from a material without any defined morphology (SrTIT-0 g) and a primary particle size of about 200 nm, the lowest PEG concentration tested induces a remarkable change in both size and shape. The material SrTIT-5 g presents a well-defined cubic shape, but also a significant increase in the size (roughly >500 nm). The cubic shape is compatible with the presence of the most stable {001} surfaces for SrTiO_3_ [30,31]. Doubling the PEG amount, the cubic microparticles disappear, leaving space to tetrahexahedron particles (cubes with oblate edges) with lower size (SrTIT-10 g, 400–500 nm). This shape is compatible with the onset of the more energetic surfaces for SrTiO_3_, {023} and {011} [30,31]. Finally, a further increase of the PEG amount leads to polyhedral particles with sizes roughly comparable to those obtained in the absence of PEG (~200 nm). These particles are obtained with a further truncation, resulting in an increased exposition of {023} and {011} facets [30,31].

**Figure 4:**
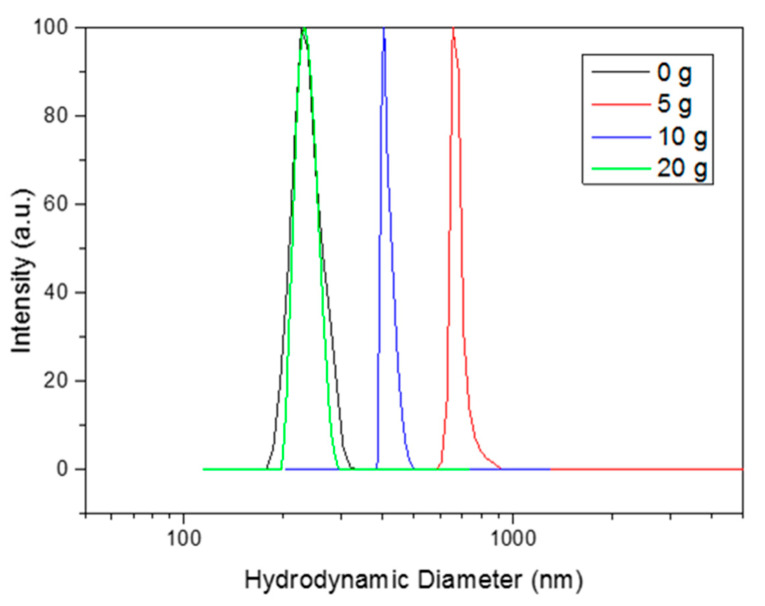
Hydrodynamic diameters (mode weighted in number of particles) of the synthesized materials measured by dynamic light scattering (DLS).

The materials were also analyzed by means of DLS to obtain further information on particle size and aggregation/agglomeration processes. Figure 4 highlights the PEG effect on the hydrodynamic diameter of the materials, confirming the effect observed via electron microscopy: a low amount of PEG (5 g) largely increases the size of the particles, while further amounts gradually decrease the size back down to the initial value of the material synthesized without a capping agent. Furthermore, the 5 and 10 g batches provide a narrower particle size distribution than the 0 and 20 g batches. This finding agrees with the SEM observations. The DLS diameter for the materials with bigger sizes (SrTIT-5 g and SrTIT-10 g) seems to be underestimated in comparison to the TEM size. This could be explained by partial sedimentation of the biggest particles, even in the presence of quite stable suspensions.

To conclude, the materials SrTIT-0 g and SrTIT-20 g have practically the same particle size; however, according to the electron microscopy, they present a different shape (Figure 3). Therefore, the comparison of these two samples allows exclusive observation of the effects of crystal morphology, without the interference of size effects.

It should be noted that in all the samples synthesized in the presence of PEG, the electron microscopy (SEM and TEM) also reveals the presence of a fraction of very fine nanoparticles (Figure 3) that are not detected in DLS measurements (Figure 4), even after sonication. This is probably due to the almost complete adhesion of these small nanoparticles to the bigger crystals, i.e., aggregation (and no agglomeration). The EDX analysis of the nanoparticles (see Figures S2–S5) highlights that these nanoparticles are characterized by aggregates of the same chemical composition as the large particles. Table 1 provides the morphological characteristics of the synthesized materials.

**Figure 5:**
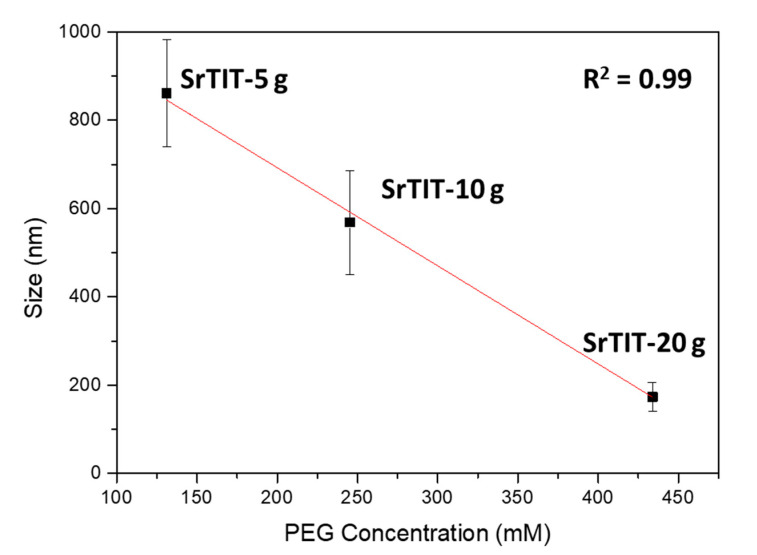
Linear dependence of the particle size on the polyethylene glycol (PEG) concentration.

The SMP size obtained from TEM analysis of the three materials synthesized in the presence of PEG follows a linear trend as a function of the shape controller concentration, as reported in Figure 5. Even though at concentrations of <100 mM this trend will be broken, because in the absence of PEG, the size is again 200 nm, this correlation provides information on the crystal growth mechanism. Within the explored concentration interval, PEG acted as a capping agent, and its interactions with the crystal surfaces not only determined the particle shape, but also progressively hindered the particle growth with increasing concentration.

The effect of PEG on the particle size is not clear. To observe such a behavior, PEG must exert at least two different competing effects and their exact nature and quantification would need further substantial investigation. Probably, PEG might induce the growth of small nuclei and then affect the aggregation level depending on its concentration.

### 3.3. UV–Vis–NIR Spectroscopy

**Figure 6:**
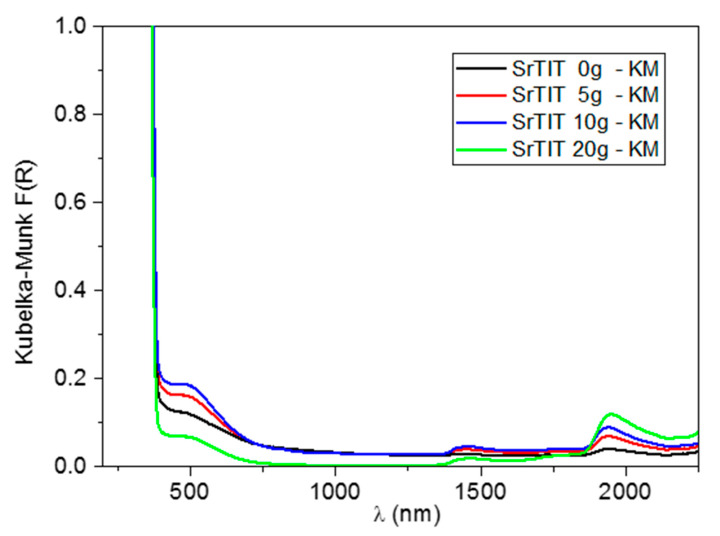
UV–vis–NIR diffuse reflectance spectra of the synthesized materials.

Figure 6 shows the UV–vis–NIR diffuse reflectance spectra of the synthesized materials. In the UV region, the spectra are dominated by the strong absorption ascribed to the band gap transition. Using a Tauc plot [32], an indirect band gap energy of about 3.2 eV can be derived for all the materials, in agreement with previously reported values [33]. Moreover, we can observe a tail in the absorption edge, extending down to 600 nm. A similar behavior has already been observed in SrTiO_3_ materials doped with nitrogen or containing oxygen vacancies [34]. However, in our case, this electronic absorption could likely be associated to the presence of traces of a lithiated phase [35] which has also been detected by XRD (see Section 2.1). Therefore, a contribution to the diffuse reflectance due to the small nanoparticles that present a defect in the amount of Sr should not be excluded.

In the NIR spectral region, we can identify the overtones and combination bands of the functional groups present on the microparticle surfaces. In particular, the broad signal centered at 1460 nm can be assigned to the overtone of a ν(OH) mode of residual polyethylene glycol and co-adsorbed water molecules [36,37,38]. The band at 1940 nm is due to the combination of the bending and asymmetric stretching modes of water molecules adsorbed on the sample surface [37,39,40]. Finally, the two weak bands at 1725 and 1760 nm are associated to the first overtone of ν(CH_2_) modes of the residual polyethylene glycol [36].

### 3.4. Photoelectrochemical Characterization

**Figure 7:**
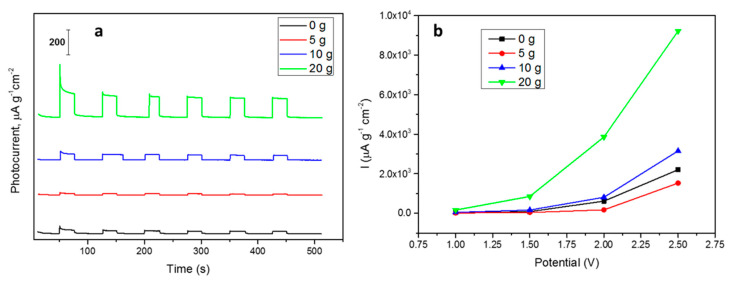
(**a**) Photocurrents measured for the synthesized materials at 1 V imposed and (**b**) trends of the photocurrents at the four imposed potentials (1, 1.5, 2 and 2.5 V).

**Figure 8:**
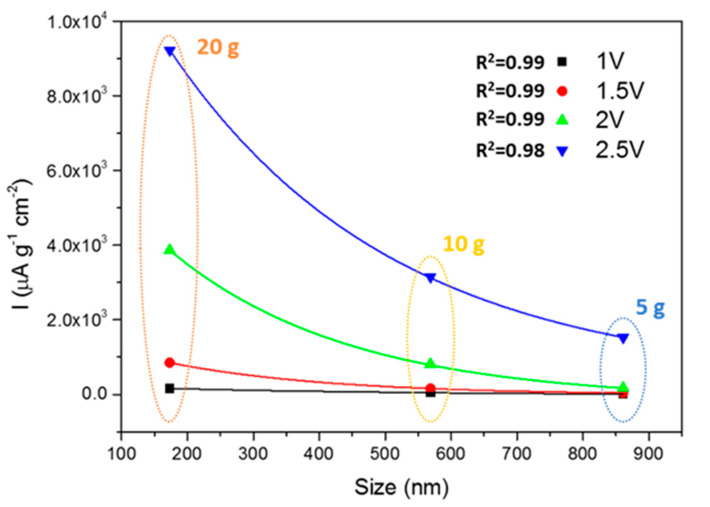
Dependence of the photocurrents from the material size for the materials obtained with PEG as a shape controller.

The photoactivities of the materials were evaluated by chronoamperometry measurements of the photocurrents (I_light_–I_dark_, Figure 7) at four different potentials (1, 1.5, 2 and 2.5 V; Figure 7). Again, there is a strong influence of both the size and shape of particles on the photoactivities. Considering the size first, and comparing the materials synthesized in the presence of PEG, it is possible to highlight an exponential-like decay of the photocurrent as the SMP size increases (Figure 8). The photocurrent can be improved in smaller semiconducting particles because the exciton generation occurs on average closer to the particle surface. This favors the surface trapping of minority carriers, whose diffusion length is usually critical, and the collection of majority carriers in the case of SrTiO_3_ electrons. This effect favors the development of larger photocurrents when smaller particles are involved. The results also highlight that the absorption due to the tail centered at 500 nm has a lower influence on the photocurrent production. In fact, the SrTIT-20 g absorption at 500 nm is the weakest, whereas its photocurrent is the largest. This means that only the UV region of the Xe lamp emission spectrum is able to produce the photocurrents.

Nevertheless, the surface chemistry is also crucial for minority carrier trapping and exciton separation, and therefore particle surface morphology must also be considered when interpreting photocurrent results. To this aim, we compared materials SrTIT-20 g and SrTIT-0 g, which had the same size, but different morphology: irregular in the case of SrTIT-0 g, while SrTIT-20 g was made of polyhedral particles exposing more regular facets. The material SrTIT-20 g significantly outperforms SrTIT-0 g, demonstrating that a defined shape is helpful for the photoactivity. These conclusions are coherent with studies in literature [30,31], where it is demonstrated that charge carrier separation is favored by the co-exposition of the oxidative surfaces and reductive surfaces. Moreover, the material SrTIT-0 g has less defined edges. As a result, almost all the particles in SrTIT-0 g present a roundish shape (Figure 3). Usually, roundish surfaces are compatible with an increase of surface defectivity, usually favoring recombination [41,42,43]. This could be the reason for the higher photoactivity of the material SrTIT-20 g. Besides surface chemistry, the different morphology could lead to a different light absorption and, therefore, an increase of the photogenerated carriers. Even in the presence of equal light extinction, the only quantity experimentally accessible, the ratio between absorption and scattering, can be significantly different. This ratio is regulated by several factors like size, shape, and agglomeration degree [44].

## 4. Conclusions

This work reports the hydrothermal synthesis of SrTiO_3_ sub-microparticles in the presence of different amounts of PEG as a capping agent. PEG greatly influences the morphology of SrTiO_3_ crystals, altering both shape and size, depending on its concentration during hydrothermal synthesis. A low amount (5 g) favors the growth of big particles with sizes above 800 nm and well-defined cuboidal shapes. Increasing the PEG concentration to 10 and 20 g, the crystals reduce their size to 570 and 175 nm, respectively. A linear dependence of the size on the PEG concentration is observed. Moreover, PEG also acts as a shape controller, synthesizing cuboid (5 g), tetrahexahedron (10 g) and polyhedral (20 g) structures, compared to the undefined (roughly cuboidal) particles obtained without PEG. The particle morphology (shape and size) considerably influences the photocurrents of the materials, significantly affecting charge carrier separation. The presence of a defined shape, together with smaller size, largely increases the photocurrent due to better charge carrier separation. Therefore, the work demonstrates the possibility to exploit PEG as a morphology controller for strontium titanate particles and once again illustrated the great importance of morphology on the photochemical behavior of crystalline semiconducting nano/microparticles.

## Figures and Tables

**Table 1:** Characteristics of the main synthesized materials.

Material	[PEG], mM	D_H_, nm	Size _TEM_, nm	Crystallographic Phase	Shape
SrTIT-0 g	0	239 ± 52	177 ± 56	SrTiO_3_	Cuboidal and undefined
SrTIT-5 g	131	680 ± 43	861 ± 122	SrTiO_3_	Cubes
SrTIT-10 g	245	417 ± 20	569 ± 118	SrTiO_3_	Tetra-hexahedron
SrTIT-20 g	434	236 ± 17	173 ± 33	SrTiO_3_	Polyhedra

## Supplementary Materials

The following are available online at https://www.mdpi.com/2079-4991/10/9/1892/s1, Figure S1: XRD Pattern of the reference JCPDS 01-089-4934; Figure S2: (A) SEM analysis of the microparticles synthetized without presence of SrTIO3 – 0 g. (B) STEM-in-SEM analysis of the microparticles synthetized without presence of SrTiO_3_ – 0 g (same field-of-view as in (A)). (C) EDX analysis of the microparticles from (A), Figure S3: (A) SEM analysis of the particles synthetized without presence of SrTiO_3_ – 5 g. B) STEM-in-SEM analysis of the same field-of-view as in (A). (C–E) EDX analysis of the particles from A, Figure S4: (A) SEM analysis of the particles synthetized without presence of SrTiO_3_ – 10 g. (B) STEM-in-SEM analysis of the same particles as in Figure A. (C–E) EDX analysis of the particles from Figure (A), Figure S5: (A) SEM analysis of the particles synthetized without presence of SrTiO_3_ – 20 g. (B) STEM-in-SEM analysis of the same field-of-view as in (A). (C–E) EDX analysis of the particles from (A).
