# Supplementary material for: Polyethylene Glycol as Shape and Size Controller for the Hydrothermal Synthesis of SrTiO3 Cubes and Polyhedra
Source: Nanomaterials (Basel). 2020 Sep 21;10(9):1892. doi: 10.3390/nano10091892 (PMC7559306; doi:10.3390/nano10091892)
Supplement: Supplementary file 1 [file nanomaterials-10-01892-s001.pdf]

nanomaterials

Supplementary Materials

Polyethylene Glycol as Shape and Size Controller for the Hydrothermal Synthesis of $SrTiO_3$ Cubes and Polyhedra

Francesco Pellegrino [1,2,*], Fabrizio Sordello [1], Lorenzo Mino [1], Marco Prozzi [1], Ulrich Mansfeld [3], Vasile-Dan Hodoroaba [3,*] and Claudio Minero [1]

[1] Dipartimento di Chimica and NIS Inter-Department Centre, University of Torino, Via P. Giuria 7, 10125 Torino, Italy; fabrizio.sordello@unito.it (F.S.); lorenzo.mino@unito.it (L.M.); marco.prozzi@unito.it (M.P.); claudio.minero@unito.it (C.M.)

[2] JointLAB UniTo-ITT Automotive, Via Quarello 15/A, 10135 Torino, Italy

[3] Federal Institute for Materials Research and Testing (BAM), 12205 Berlin, Germany; Ulrich.Mansfeld@uni-bayreuth.de

* Correspondence: Francesco.pellegrino@unito.it (F.P.); dan.hodoroaba@bam.de (V.-D.H.)

X-Ray Diffraction Reference Pattern

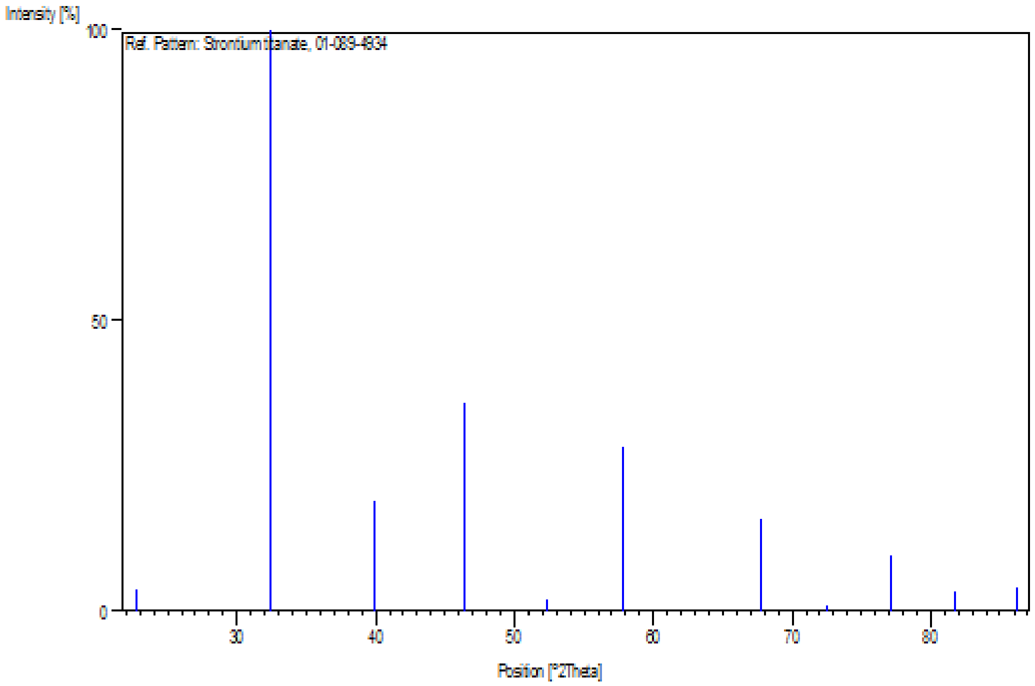

Figure S1. XRD Pattern of the reference JCPDS 01-089-4934.

In Figure S1 is reported the XRD Pattern of the reference JCPDS 01-089-4934, strontium titanate.

EDX Mapping Analysis

- **SrTIT 0 g**

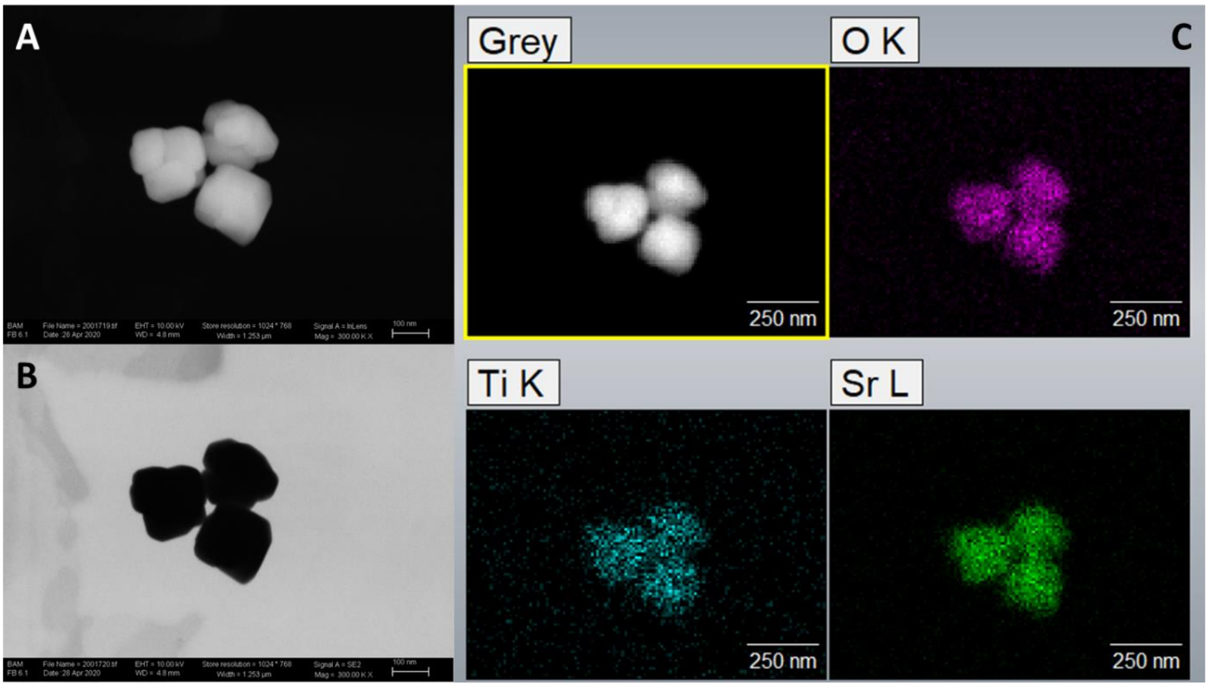

Figure 2. (A) SEM analysis of the microparticles synthetized without presence of $SrTiO_3$ – 0 g. **(B)** STEM-in-SEM analysis of the microparticles synthetized without presence of $SrTiO_3$ – 0 g (same field-of-view as in **(A)**). **(C)** EDX analysis of the microparticles from **(A)**.

Figure S2 highlights that the synthetized particles are $SrTiO_3$. No fine nanoparticles (as in the case of the other materials) were detected.

- **SrTIT 5 g**

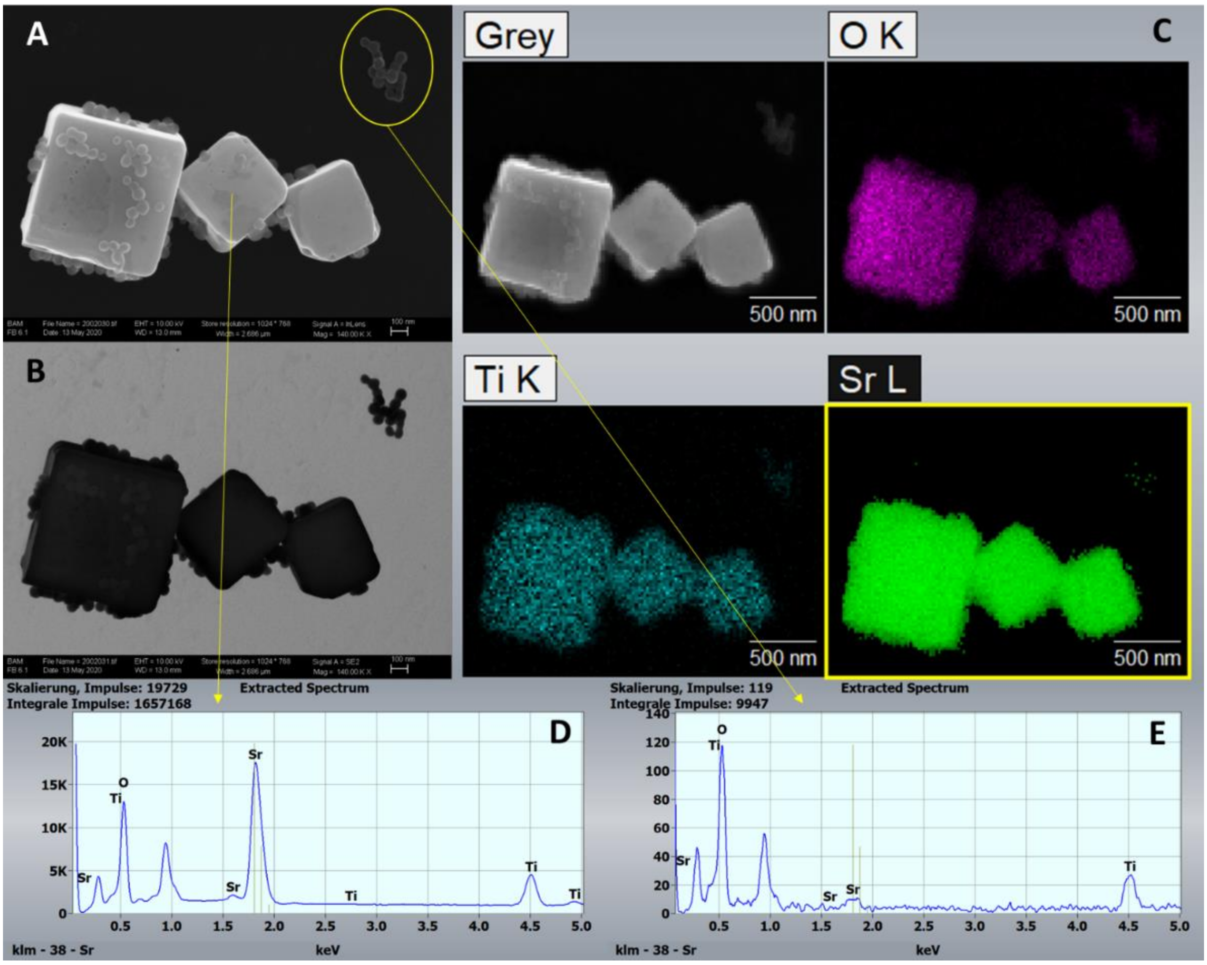

Figure 3. (A) SEM analysis of the particles synthetized without presence of $SrTiO_3$ – 5 g. **(B)** STEM-in-SEM analysis of the same field-of-view as in **(A)**. **(C–E)** EDX analysis of the particles from **(A)**.

Figure S3 highlights that the synthetized particles are $SrTiO_3$. Note that the isolated fine nanoparticles marked with the yellow circle (top-right) could be analyzed by high-resolution EDX and all elements Ti, Sr and O are visible. As described in the main manuscript, a quantification of the EDS spectra of particles well below µm (with surface morphologies deviating strongly from planar) is not possible with the conventional µm-bulk EDS approaches. Even if the Sr signal for the fine nanoparticles is lower relative to the large particles, this is a clear indication of Sr titanate and excludes the presence of particles such as e.g., Ti oxide or pure Sr oxide. In other words, both types of particles, the large and fine ones, are of the same material. Isolated fine particles have been found as very rare, vast majority of small particles are aggregated to the large particle.

- **SrTIT 10 g**

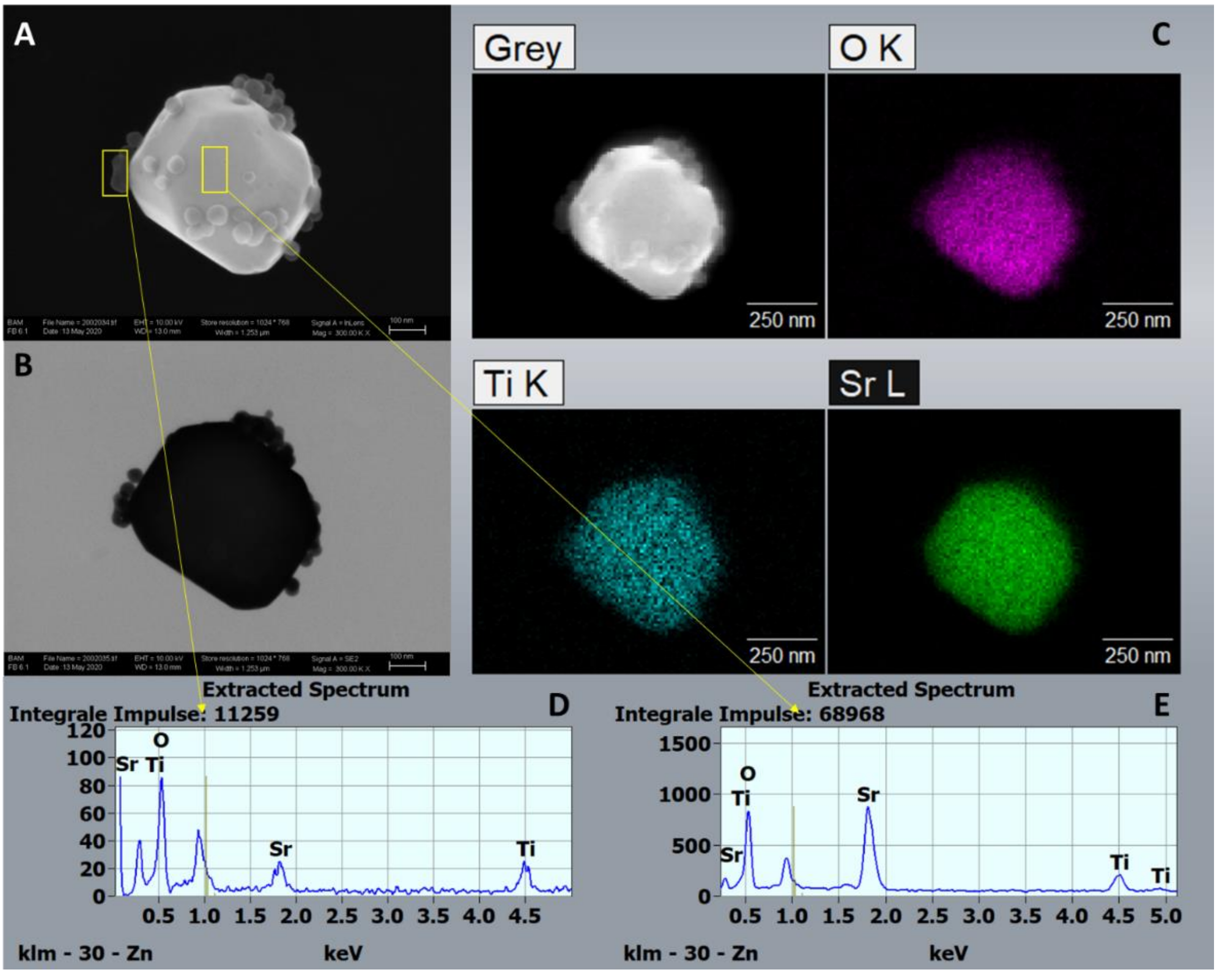

Figure 4. (A) SEM analysis of the particles synthetized without presence of $SrTiO_3$ – 10 g. **(B)** STEM-in-SEM analysis of the same particles as in Figure **(A)**. **(C–E)** EDX analysis of the particles from Figure **(A)**.

Figure S4 highlights that the synthetized particles are $SrTiO_3$. As mentioned in the main text, the nanoparticles present at the surface of the $SrTiO_3$ microparticles were characterized by aggregates of same chemical composition, and similar to the matrix constitution of previous material.

- **Sr TIT 20 g**

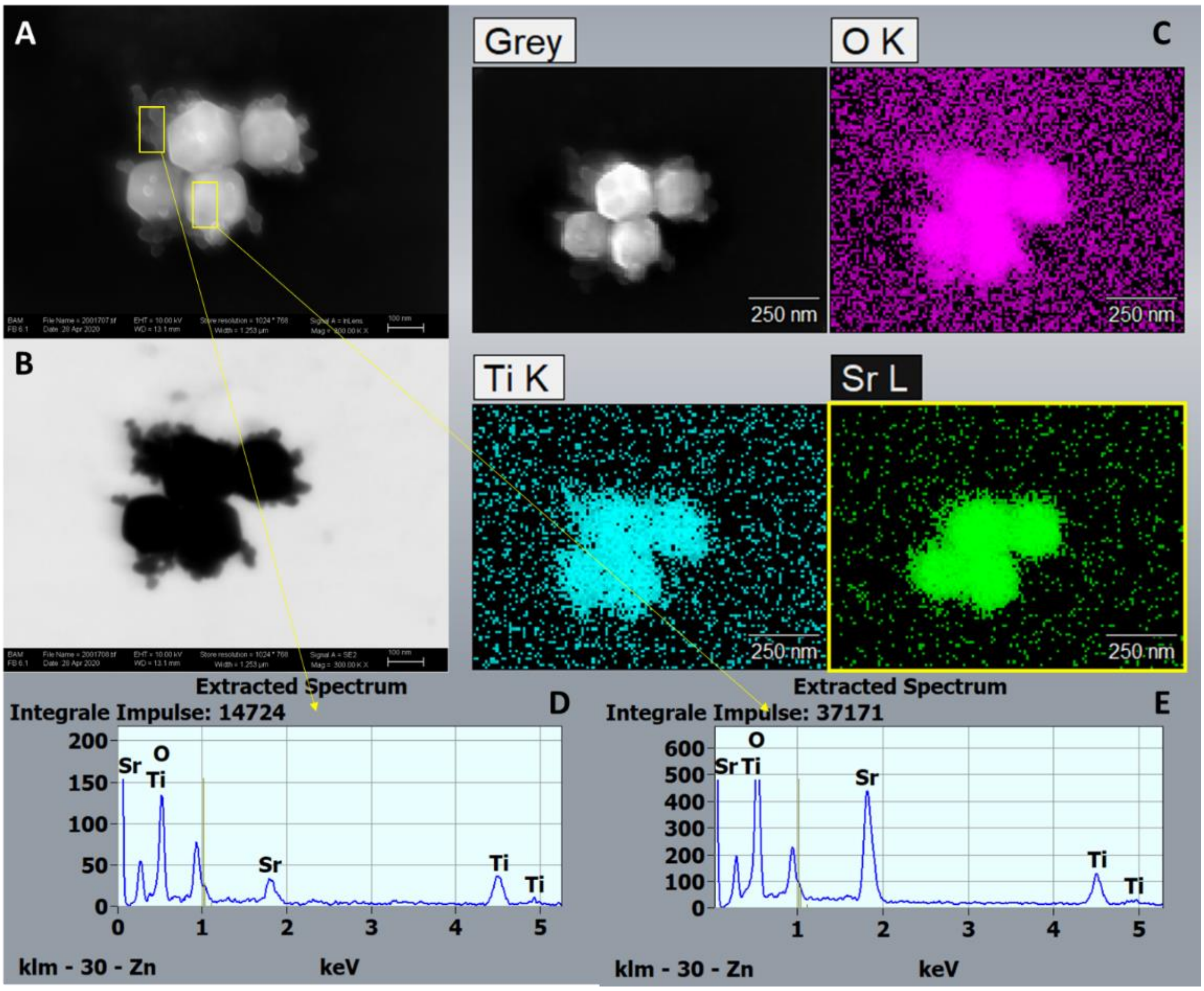

Figure 5. (A) SEM analysis of the particles synthetized without presence of $SrTiO_3$ – 20 g. **(B)** STEM-in-SEM analysis of the same field-of-view as in **(A)**. **(C–E)** EDX analysis of the particles from **(A)**.

Figure S5 highlights that the synthetized particles are $SrTiO_3$. As mentioned in the main text, the nanoparticles present at the surface of the $SrTiO_3$ microparticles were characterized as aggregated fine nanoparticles of similar chemical composition as the large particles.
